# A novel technology to integrate imaging and clinical markers for non-invasive diagnosis of lung cancer

**DOI:** 10.1038/s41598-021-83907-5

**Published:** 2021-02-25

**Authors:** Ahmed Shaffie, Ahmed Soliman, Xiao-An Fu, Michael Nantz, Guruprasad Giridharan, Victor van Berkel, Hadil Abu Khalifeh, Mohammed Ghazal, Adel Elmaghraby, Ayman El-baz

**Affiliations:** 1 BioImaging Laboratory, Department of Bioengineering, University of Louisville, Louisville, KY USA; 2 Department of Chemical Engineering, University of Louisville, Louisville, KY USA; 3 Department of Chemistry, University of Louisville, Louisville, KY USA; 4 Department of Cardiovascular and Thoracic Surgery, University of Louisville, Louisville, KY USA; 5 Chemical Engineering Department, Abu Dhabi University, Abu Dhabi, UAE; 6 Department of Electrical and Computer Engineering, Abu Dhabi University, Abu Dhabi, UAE; 7 Computer Science and Engineering Department, University of Louisville, Louisville, KY USA

**Keywords:** Cancer, Biomarkers, Lung cancer

## Abstract

This study presents a non-invasive, automated, clinical diagnostic system for early diagnosis of lung cancer that integrates imaging data from a single computed tomography scan and breath bio-markers obtained from a single exhaled breath to quickly and accurately classify lung nodules. CT imaging and breath volatile organic compounds data were collected from 47 patients. Spherical Harmonics-based shape features to quantify the shape complexity of the pulmonary nodules, 7th-Order Markov Gibbs Random Field based appearance model to describe the spatial non-homogeneities in the pulmonary nodule, and volumetric features (size) of pulmonary nodules were calculated from CT images. 27 VOCs in exhaled breath were captured by a micro-reactor approach and quantied using mass spectrometry. CT and breath markers were input into a deep-learning autoencoder classifier with a leave-one-subject-out cross validation for nodule classification. To mitigate the limitation of a small sample size and validate the methodology for individual markers, retrospective CT scans from 467 patients with 727 pulmonary nodules, and breath samples from 504 patients were analyzed. The CAD system achieved 97.8% accuracy, 97.3% sensitivity, 100% specificity, and 99.1% area under curve in classifying pulmonary nodules.

## Introduction

In 2019, there were approximately 234, 030 new cases of lung cancer and 154, 050 related deaths^1^. Early diagnosis of lung cancer significantly improves the effectiveness of treatment and increases the five-year survival rate from $17.7\%$ to $55.2\%$ ^2–4^. Further, it has been demonstrated that patients with smaller, early stage tumors have a much higher survival rate than patients with larger than T1 tumors^3,5^. Current lung cancer screening methodologies can reduce lung cancer mortality by up to $20\%$ if implemented appropriately, but currently, only $32\%$ of patients diagnosed with lung cancer are at an early stage (Stage I or II)^1^. Non-invasive diagnosis of lung cancer is currently accomplished through imaging techniques.

### Imaging markers

The advent of CT scanning has enabled large-scale screening for lung cancer. The National Lung Screening Trial (2011) detected a high proportion of early cancers ($49\%$ stage IA) using CT scans, allowing for intervention with curable intent, which resulted in a $20\%$ reduction in lung cancer mortality^6^. However, while only $1.1\%$ of the patients were found to have a malignancy in the screening arm, $27\%$ of patients had a positive finding on their screening CT scan. These false positive cases were primarily benign pulmonary nodules that required further investigations including serial CT scanning, positron emission tomography (PET), bronchoscopy, percutaneous biopsy or surgical intervention for the correct diagnosis. Sequential CT examining to watch growth, or texture changing is commonly utilized for sub-centimeter nodules, which takes up to two years of follow-up for lung cancer detection. The prolonged follow-up period may reduce patient compliance, delay diagnosis and delay treatment, which increases treatment costs and decreases lung cancer survival rate. For nodules that are larger than 8 mm, PET scans of chest may be utilized to predict the likelihood of its malignancy. The main disadvantages of the PET scans is its high false positive specially for juxtapleural pulmonary nodules. These factors were the real reason behind expanding the clinical diagnosis suspicion of lung cancer and increasing the need for surgical examination through biopsies to set aside the malignancy. Bronchoscopy and percutaneous biopsies are still the most reliable way for diagnosis but there is a real need to eliminate the risk associated with this surgical procedure, especially when the malignancy likelihood is not high, as the surgical interaction for the benign nodule is considered a clinical failure because the benign nodules do not have any risk or cause any harm to the patient. The prohibitive costs associated with repeated radiographic scans and the morbidity due to unnecessary invasive procedures for benign nodules necessitate the development of new diagnostic modality that can detect malignant pulmonary nodules (lung cancer). In an imaging-based CAD system, nodule detection and nodule classification are distinct but essential components. Nodule detection only detects and segments the nodule and provides no information on the malignancy of nodules^7–9^. Automatic nodule detection and segmentation techniques have been previously described and implemented by several groups, including our group^7–11^. Accurate nodule classification determines if the nodule is malignant or benign, which is challenging but essential for cancer diagnosis. The primary focus of this manuscript is the nodule classification using data from a single CT scan and an inexpensive breath test. Currently, various computational methods exist for classification of lung nodules detected in multiple, serial CT scans^12–17^. However, despite requiring multiple serial CT scans for indeterminant pulmonary nodules over two years, these methods have a low classification accuracy for early diagnoses of lung cancer because they: (1) do not account for large deformations in lung tissue due to breathing and beating of the native heart; and (2) do not use the 3D shape and appearance of detected nodules in conjunction with estimated nodule growth rate. Importantly, these methods are unsuitable for certain types of lung nodules (e.g. cavities and ground glass nodules), and are difficult for clinical practitioners to use as it requires significant graphic interaction.

### Clinical bio-markers

Detection of lung cancer bio-markers from saliva, urine, blood, and exhaled breath of patients is a developing modality for non-invasive diagnosis. Li et al.^18^ demonstrated that genetic deletions of HYAL2, FHIT, and SFTP in saliva can be used as diagnostic markers for non-small cell lung cancer (NSCLC). LRG1 has been proposed as a candidate bio-marker for diagnosis of NSCLC in urine^18^. Oxidative stress produced by the variable redox environment within cancer is thought to increase the production of various volatile organic compounds (VOCs). Hanai et al.^19^ used the urinary VOCs to potentially identify lung cancer. Begum et al.^20^ identified six genes (APC, CDH1, MGMT, DCC, RASSF1A, and AIM1) in blood which could be used as a bio-marker for lung cancer diagnosis. Antibodies in patient blood has also been proposed as a bio-marker for lung cancer in an early stage^21^. Early diagnosis of lung cancer using quantitative analysis of carbonyl VOCs in exhaled breath has been recently reported^22–26^. Analysis of bio-markers is usually quantitative and inexpensive. However, despite three decades of research and thousands of reports of bio-markers, very few bio-markers have established clinical utility. The diagnostic usefulness of imaging modalities and bio-markers remain limited as the accuracy, sensitivity, and specificity of these bio-markers typically do not exceed $80\%$, which is lower than thresholds required for reliable diagnosis $(>95\%)$. Thus, the objective of this study is to develop and test a clinical diagnostic tool that integrates patient breath bio-marker data with novel image-based CT markers to improve accuracy and speed of lung cancer diagnosis. To the best of our knowledge, our approach is the first to combine both breath test bio-markers and imaging markers for early diagnosis of lung cancer. The proposed CAD system is non-invasive, requiring only a single CT scan and a breath test to rapidly and accurately diagnose lung cancer (a few days compared to two years), with the potential to greatly reduce lung cancer diagnosis costs and increase the patient survival rate.

## Materials and methods

### Patients

CT and breath analysis data were both collected on the same day for every patient from 47 patients in the period from 2016 to 2018 (Tables 1,2). Our collaborators at the university of Louisville hospital recruited patients with age ranges from 40 to 90 years and collected both a CT scan and a breath test (the diagnosis for most of these patients is biopsy confirmed). Retrospective analyses have an inherent risk of selection bias, despite our inclusion criteria not having any demographic filters that might introduce bias. The research protocol was approved by the Institutional Review Board (IRB) at the University of Louisville and all methods were performed in accordance with the relevant guidelines and regulations. After the patient informed consent was obtained, one liter of mixed tidal and alveolar breath sample was collected into a non-reactive Tedlar bag (Sigma Aldrich, St Louis, Mo) from a single exhalation from each participant^23^. The CT data was collected from the same 47 patients after obtaining the patient informed consent also with a slice thickness of 2.5 mm reconstructed every 1.5 mm, KV 140, MA 100, and F.O.V 36 cm. The ground truth for nodule detection and segmentation was obtained by the union of the masks of nodules that were manually segmented by three radiologists that have the same level of knowledge (greater than 10 years’ experience) and there was no questionable difference between their final decisions. Patient selection was blinded but included patients with both benign and malignant small lung nodules (4 to 20 mm) and large nodules ($> 20\,{\text{mm}}$). The patient diagnostic conclusions from the radiologists were blinded from the data analysis team for lung cancer diagnosis using both breath test and CT markers. The patients were either biopsied for diagnostic conclusion (these patients do not need follow-up) or followed for up to two years until a final lung cancer diagnosis could be determined based on current clinical approaches (serial CT scans every 6 months and/or biopsy/bronchoscopy). If there was no change in the CT scan over two years, the nodule was considered benign. The accuracy, sensitivity, and specificity of the proposed CAD system were determined based on the final lung cancer diagnosis using conventional clinical methods (ground truth).

**Table 1:** Demographics and nodule size of the patients ($\hbox {n}=47$ patients). $\hbox {D}= nodule$ diameter.

	Subject	Male	Female	Nodule size
Malignant	20	3	17	$4\,{\text{mm}}\le D \le 20\,{\text{mm}}$
	17	9	8	$20\,{\text{mm}}\le D \le 60\,{\text{mm}}$
Benign	5	1	4	$4\,{\text{mm}}\le D \le 20\,{\text{mm}}$
	5	5	0	$20\,{\text{mm}}\le D \le 34\,{\text{mm}}$

**Table 2:** Clinical characteristics of the patients.

	All patients ($\hbox {N}=47$)	Male ($\hbox {N}=18$)	Female ($\hbox {N}=29$)
Age (years)	48–93	59–93	48–88
Malignant	37	12	25
White race	30	11	19
Height (cm)	152–188	170–188	152–180
Weight (Kg)	39–168	61–156	39–168
Active smoker	21	10	11
Previous smoker	20	7	13
Lifelong non-smoker	6	1	5
Personal history of lung cancer	7	3	4
Personal history of any cancer	19	8	11

The sample size of patients with both breath and imaging data was limited. To mitigate this limitation and validate the classification methodology, we used retrospective CT scans from 467 patients with 727 nodules ($\hbox {benign}=413$, malignant $=314$) from the Lung Image Database Consortium (LIDC) database^27^. The nodules were detected, delineated, and diagnosed by four radiologists, where each of them assigned a malignancy score on scale of 1 to 5 (1 represents benign and 5 represents malignant). Although the LIDC database contains 1018 patients, we used only 727 nodules which had a high degree of confidence and agreement between the four radiologists. Specifically, only nodules that received an average score of 3.5 or greater (deemed malignant) and nodules with an average score of 1.5 or lower (deemed benign), were included in this study. For breath analysis, samples from 504 patients were collected ($\hbox {benign}=252$, $\hbox {malignant}=252$) and analyzed. The malignant nodules were confirmed by pathological diagnosis and the benign ones were confirmed by tissue diagnosis or repeated CT scans with no discernible change or decrease in size for $\ge 2$ years.

### Computer aided diagnostic system for nodule classification

**Figure 1:**
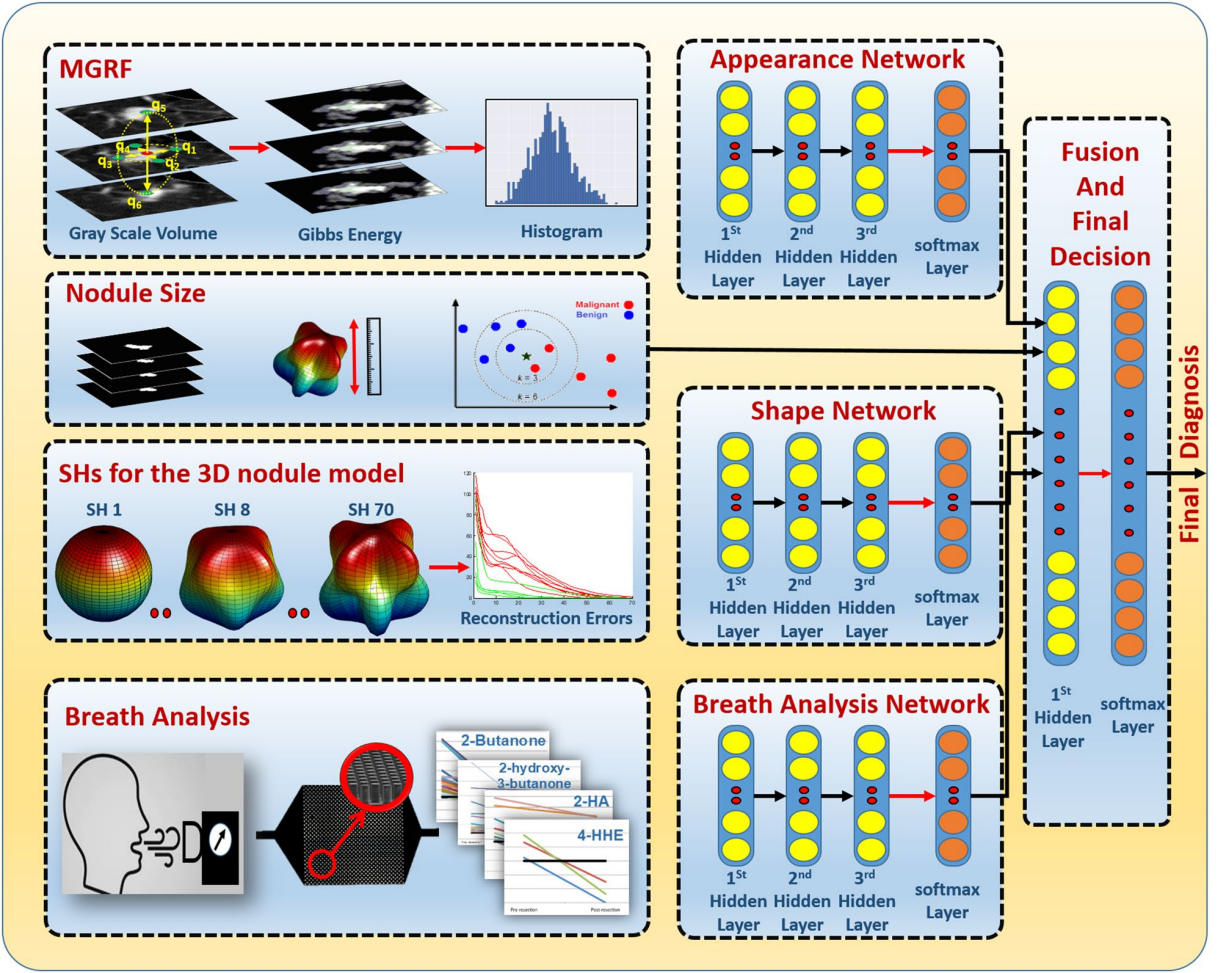
Lung nodule classification framework.The framework was generated by Microsoft PowerPoint 2019 (https://www.microsoft.com/zh-cn/microsoft-365/powerpoint).

The CAD system integrates data from a single CT scan for computed tomography markers and a single breath test for cancer bio-markers for classification of lung nodules. The methodology of obtaining imaging markers, breath bio-markers, and integration of breath and imaging markers are presented next (see Fig. 1 for the framework).

### Imaging markers from CT data

Size, three-dimensional (3D) shape, and 3D appearance analyses were performed on the CT images from the clinical data ($\hbox {n}=47$).

#### Size analysis

Larger sized nodules tend to be malignant^28^. Classification based on nodule size, while straightforward, does not lend itself to high levels of classification accuracy for smaller sized nodules. A basic K-NN classifier was fed with the nodules’ size data and was used to give an initial malignancy probability for each pulmonary nodule.

#### Shape analysis

Malignant nodules grow faster than benign nodules and thus have a more complex shape and surface. Surface shape complexity was quantified using spherical harmonic (SH) decomposition^29^. Malignant nodules with complex surfaces require more SHs than the smoother benign nodules, enabling classification between malignant and benign nodules. Briefly, a spectral SH analysis was used to model the pulmonary nodules, by considering its surface as a linear combination of particular basis functions. After the triangulated 3D mesh is built, it is mapped to the unit sphere for the SH decomposition. A new mapping approach, the Attraction-Repulsion Algorithm, was developed to ensure that: (i) the distance from the center of the nodule to any node as unity, and (ii) each node is equidistant to all its neighbors.

Let I refer to the number of mesh nodes, the cycle iterator, and $C_{\alpha ,i}$ the coordinates of node *i* at cycle number $\alpha$. Let *J* represent the number of neighbors for the mesh node and $d_{\alpha ,ij}$ denote the Euclidean distance between *i* and *j* at cycle number $\alpha$, where $j = 1,...,J$. Let $d_{\alpha ,ij}=C_{\alpha ,j}-C_{\alpha ,i}$ denote the displacement between the nodes *j* and *i* at cycle number $\alpha$. Let $C_{A,1}$, $C_{A,2}$, $C_{R}$ be the constants controlling the displacement for each surface node. The attraction step adjusts the location for each node $C_{i}$ to be centered with respect to its neighbors and is given by:

1 $$ {\mathbf{C}}_{{\alpha ,i}}^{\prime }  = {\mathbf{C}}_{{\alpha ,i}}  + C_{{{\text{A}},1}} \sum\limits_{{j = 1;j \ne i}}^{J} {{\mathbf{d}}_{{\alpha ,ji}} d_{{\alpha ,ji}}^{2}  + C_{{{\text{A}},2}} \frac{{{\mathbf{d}}_{{\alpha ,ji}} }}{{d_{{\alpha ,ji}} }}}  $$

**Figure 2:**
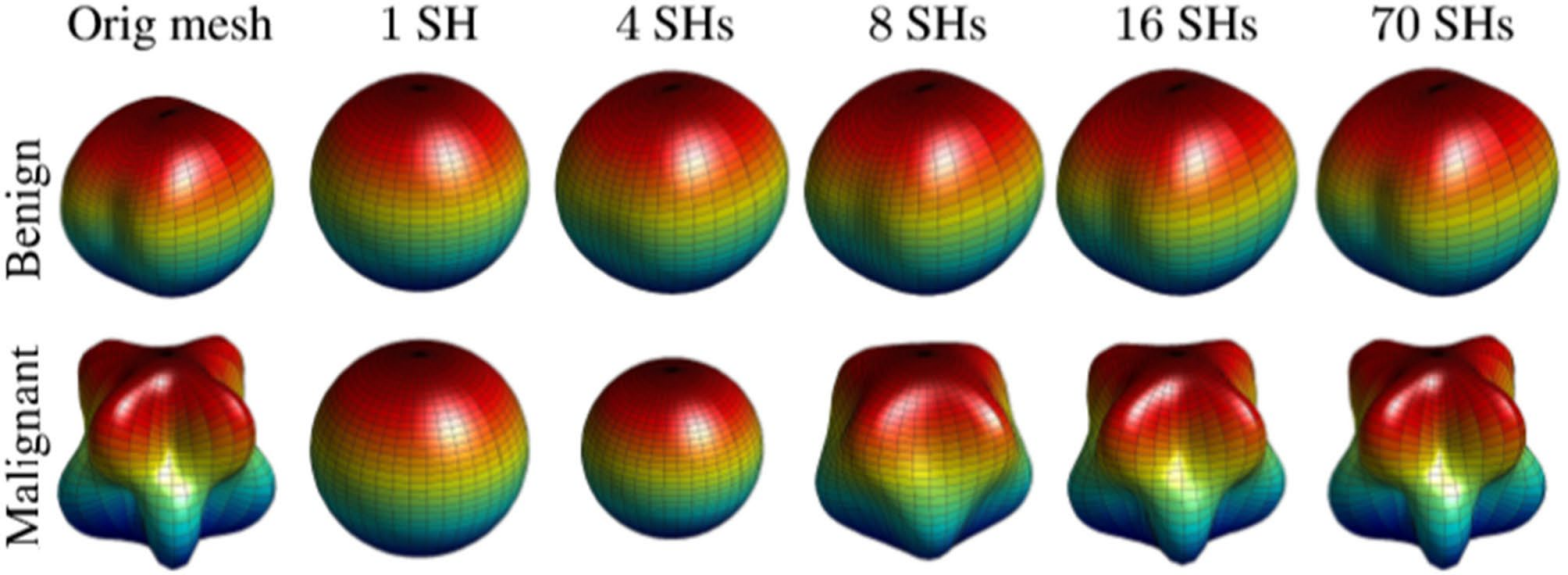
Shape approximation for malignant and benign nodules.The figure was created in MATLAB R2018B (https://www.mathworks.com/products/matlab.html).

The nearer nodes are pushed further from each other, while $C_{A,2}$ keeps the nodes from collapsing. Thus, the entire mesh is inated in the repulsion step by pushing every node outward to preserve the equidistant condition after their last back-projection onto the unit sphere along the rays from the sphere’s centroid. In the repulsion step, every node is pushed outward to maintain the equidistant condition after their last back-projection onto the unit sphere along the rays from the sphere’s centroid. To avoid overlap or crossing over of nodes during shifting, the location for each $C_{i}$ is updated after the back-projection as:

2 $$\begin{aligned} C^o_{\alpha +1,i}={\acute{C}}_{\alpha ,i}+\frac{C_R}{2I}\mathrm {\ } \sum ^I_{j=1;j\ne i}{\left( \frac{{\mathrm {d}}_{\alpha ,ji}}{{\left| d_{\alpha ,ji}\right| }^2}\right) }\mathrm {\ } \end{aligned}$$

where $C_{R}$ is the repulsion constant. After the mapping process, the nodule surface was approximated by a linear combination of SHs. Lower-order harmonics will be adequate to approximate a more uniform shape (benign nodules), compared to higher-order harmonics for more complex shapes (malignant nodules), Fig. 2. The SHs coecients from up to 70 harmonics were subsequently used to reconstruct the original pulmonary nodule.

#### Appearance analysis

Malignant nodules, due to their high growth rate, have a non-uniform density (spatial non-homogeneity) compared to benign nodules, which is reflected as varying Hounsfield units (HU) in the CT scan. Hounsfield units are a unit of measure that represents the different density levels of tissues as visualized in the CT images. The Appearance analysis is modeled for the 3D nodule volumes in a way that the differences between the HU of a voxel and its 7 nearest neighbors is represented as Gibb’s energy using a 7th order Markov Gibbs Random Field (MGRF). This model tackles the inherited challenges within the CT images that stem from partial volume effect, different acquisition parameters, and scanner types while preserving ordinal signal relations to keep the visual appearance. Besides, the 7th-order MGRF model uses the partial ordinal interaction instead of the complete ordinal ones to reduce the cardinality and makes the model more computationally feasible. Grayscale patterns of the nodules are considered as samples of a trainable translation- and contrast-oset-invariant 7th-order MGRF.

This model relates the relation between the Gibbs energy, $E_7 (g)$, voxel-wise HU, g(r), and an image texture, $g=(g(r):r\in \mathbb {R})$ in a general-case exponential family distribution as follows:

3 $$\begin{aligned} P_z\left( \varvec{\mathrm {g}}\right) = \left. \frac{1}{Z} \mathrm {\ exp}\left( -\sum _{a=1}^{A}\sum _{\varvec{\mathrm {\mathbf {c}}}_{\mathrm {a:r}}\in \mathbb {C}_a} \mathrm {\mathbf {V}_a}\left( g(\mathrm {\mathbf {r'}}):\mathrm {\mathbf {r'}}\in \mathrm {\mathbf {c}}_{a:r}\right) \right) \right) \end{aligned}$$

Where the Gibbs energy $E_7 (g) = \sum _{\varvec{\mathrm {\mathbf {c}}}_{\mathrm {a:r}}\in \mathbb {C}_a} \mathrm {\mathbf {V}_a}(g(\mathrm {\mathbf {r'}}):\mathrm {\mathbf {r'}}\in \mathrm {\mathbf {c}}_{a:r}),$ and the function *Z* normalizes the distribution over the parent population $Z = \sum \nolimits _{g\in \mathbb {G}}{\exp {(-E(\mathbf {g}))}}$, and the interaction structure is a system, $\mathbb {C}$, of $A, A\ge 1$, clique families, $\mathbb {C}_{a}$. The origin voxel, $\mathbf {r}\in \mathbb {R}$ and a *K*-variant Gibbs potential function $\mathbf {V}_a(g(\mathrm {\mathbf {r'}}): \mathrm {\mathbf {r'}}\in \mathrm {\mathbf {c}}_{a:r})$ depends on the ordinal relationships between the origin voxel and the 7 neighbours, $\mathbf {\mathrm {r'}}\in \mathrm {\mathbf {c}}_{a:\mathbf {\mathrm {r}}}; \mathrm {\mathbf {r'}}\ne \mathrm {r}$.

The signal interactions are modeled between each voxel and the 7 neighbors at a distance, $\rho$, from that voxel. The Gibbs potentials of the 7-voxel subsets, are learned from the training nodules, $g^{\circ }$, to be used in computing the energy $E_7(g)$. The learning process uses the maximum likelihood estimates (MLE) that generalize the analytical approximations of the $2^{nd}$-order MGRF potentials in^30^:

4 $$\begin{aligned} {\upsilon }_{7:\rho }\left( \beta \right) =\frac{F_{7:\rho :core}\left( \beta \right) -F_{7:\rho }\left( \beta :{\mathrm {g}}^o\right) }{F_{7:\rho :core}\left( \beta \right) (1-F_{7:\rho :core}\left( \beta \right) )}\ ;\ \beta \in {\mathbb {B}}_7 \end{aligned}$$

**Figure 3:**
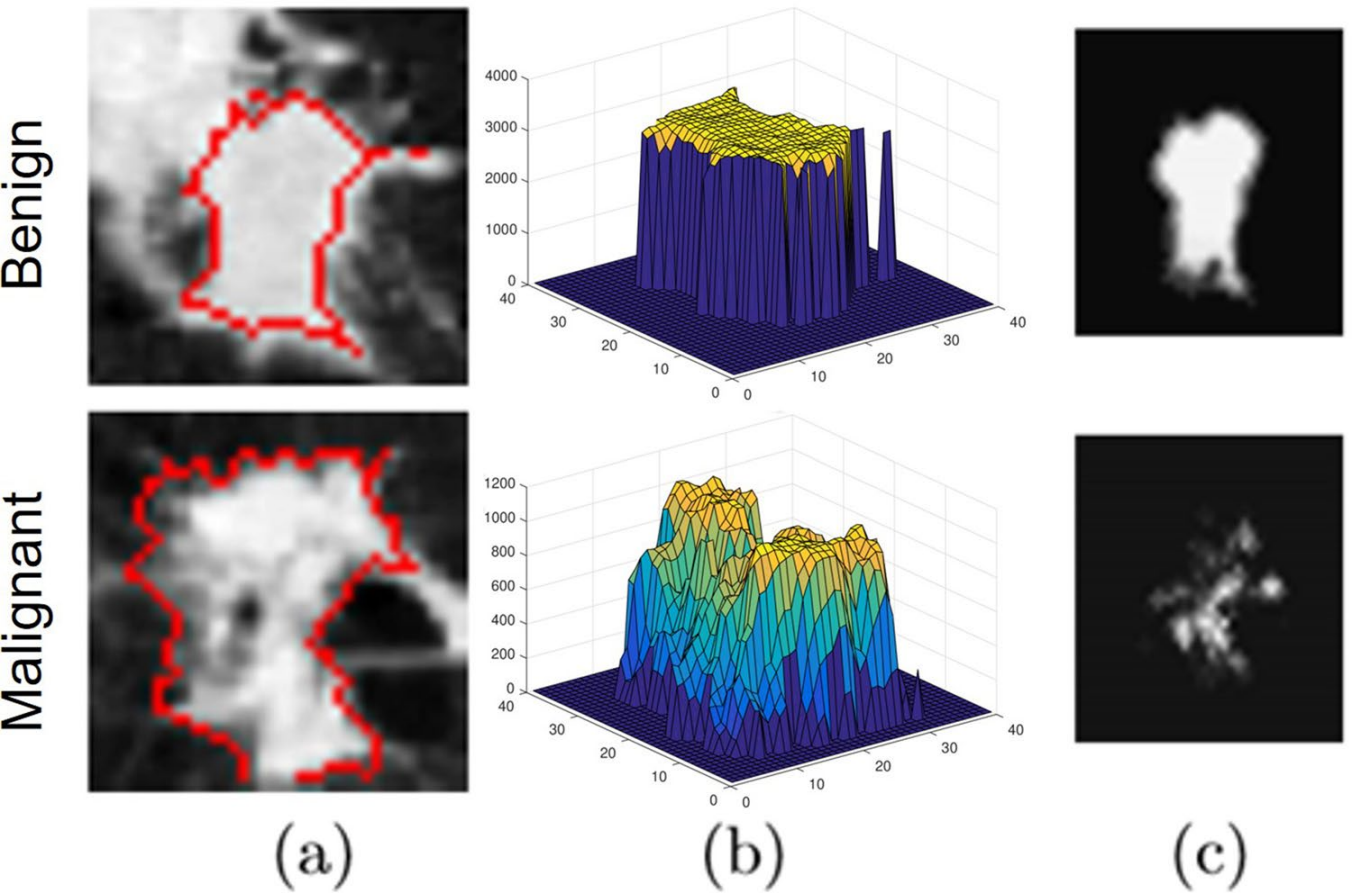
A sample of benign (rst) and malignant (second-row) nodules (**a**), their 3D visualization of HU values (**b**), and their Gibbs energy which shows high energy for (brighter) for benign and less energy for malignant (darker) (**c**). The figure was created in MATLAB R2018B (https://www.mathworks.com/products/matlab.html).

Here, $\beta$ is a coded contrast-offset-invariant relation between the seven signals; $\mathbb {B}_7$ denotes the set of codes for the possible ordinal 7-signal relations; $F_{7:\rho }\left( {\mathrm {g}}^o\right)$ is an empirical marginal probability of the code $\beta$ ; $\beta \in \mathbb {B}_7$, over all the 7-voxel congurations with the center-to-voxel distance $\rho$ in $\mathrm {g}^o$, and $F_{7:\rho :core}\left( \beta \right)$ is the like probability for the core distribution. The computed energy is used as a descriptive feature to discriminate between the malignant and benign nodules (Fig. 3).

The training nodules, $\mathrm {g}^o$, are used to learn both the potentials and the distance $\rho$ between the central voxel and its neighbors. The output features from the MGRF appearance model is a vector of size 1000 describing the histogram bins of the Gibbs energy for each nodule.

### Breath bio-markers

**Figure 4:**
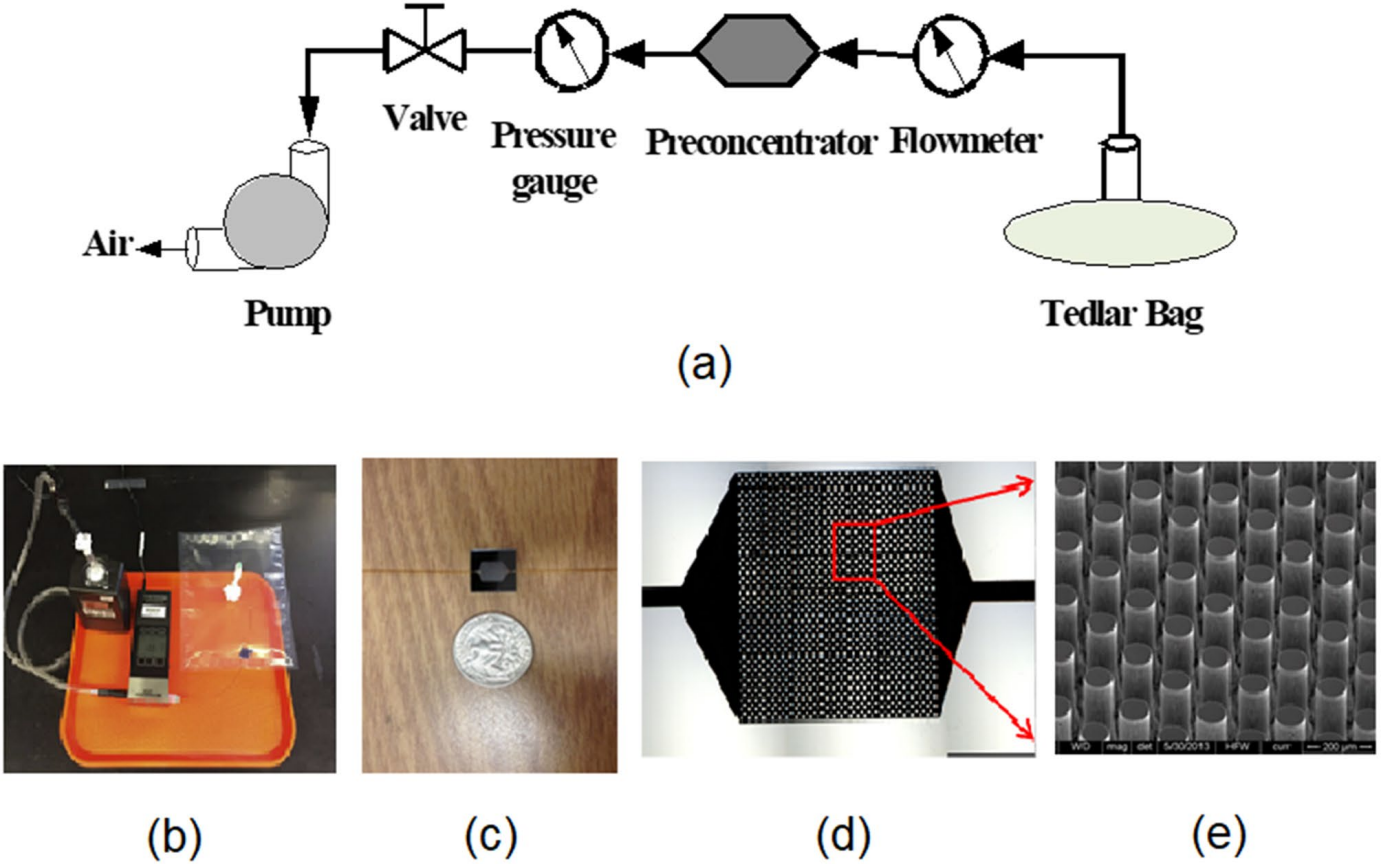
(**a**) Schematic setup for capture of carbonyl VOCs in exhaled breath, (**b**) photo of the breath collection system, (**c**) A microfabricated microchip with fused silica tubes attached to inlet and outlet ports; (**d**) optical picture of the microchip created by DRIE; (**e**) SEM micrograph of the micropillar array within the preconcentrator.

Quantification of carbonyl VOCs: The exhaled breath collected in 1-L Tedlar bags were drawn through a proprietary microreactor chip by applying a vacuum (Fig. 4). The surfaces of micropillars of the microreactor chip are coated by 2-(aminooxy)-N, N, N-trimethylethanammonium (ATM) iodide^22^. ATM chemoselectively traps carbonyl compounds in exhaled breath by means of oximation reactions. After the breath sample was completely evacuated from the Tedlar bag, ATM adducts in the microreactor chip were eluted with 100 mL of methanol from a slightly pressurized small vial. The eluted solution was analyzed directly by Fourier transform-ion cyclotron resonance mass spectrometry (FT-ICR-MS)^22^. FT-ICR-MS is a hybrid linear ion trap MS (Finnigan LTQ FT, Thermo Electron, Bremen, Germany) equipped with a TriVersaNanoMate ion source (AdvionBioSciences, Ithaca, NY) with an electrospray chip (nozzle inner diameter 5.5 mm) that was used to analyze all breath samples using the eluted solution. A known amount of deuterated acetone completely reacted with ATM (ATM-acetone-d6) in methanol was added to the eluted solution as internal reference for quantification of ATM adducts. The concentrations of all 27 carbonyl VOCs detected in exhaled breath were determined by comparison of the relative abundance with that of added ATMacetone-d6.

### Nodule classification

In order to diagnose the lung nodules, a deep neural network with stacked autoencoder (AE) was used. Three auto-encoder-based classifiers, one classier each for shape, appearance, and exhaled breath compounds, were utilized to give an initial estimation for probabilities of the classification, which are axed together with the probabilities of the k-NN classier for size. The axed probabilities were then input into the second stage auto-encoder to give the final classification of nodules (see Fig. 1 for more details). AE was utilized to decrease the dimensionality of the features with three-layered neural networks to identify the most distinguishable features by pre-training algorithm. All the hidden layers reduced the hidden shape descriptors from 70 (corresponding to 70 SHs) to 10, hidden appearance descriptors from 1000 (corresponding to 1000 histogram bins for Gibb’s energy) to 100, and 27 hidden breath descriptors (corresponding to 27 VOCs) to 10. After the AE layers, a softmax layer was used to boost the diagnosis accuracy by limiting the overall loss of the labeled data during the training.

Briefly, for each AE, let $W=\{W_j^e; W_i^d :j=1,...,s ;i=1,...,n\}$ refer to a set of weights column vectors for encoding, *E*, and decoding, *D*, layers, and let *T* denote vector transposition. The AE alters the n-dimensional column vector $u=[u_1,...,u_n]^T$ into an s-dimensional column vector $h=[h_1,...,h_s]^T$ of level activators such that $s < n$ by nonlinear uniform transformation of *s* weighted linear combinations of input as:

$h_j=\sigma ((W_j^e)^{T}u)$, where $\sigma (.)$ is a sigmoid function with values from [0,1]

5 $$\begin{aligned} \sigma (t)=\frac{1}{1+e^{-t}} \end{aligned}$$

The softmax layer calculates the classication probability through the following equation:

6 $$\begin{aligned} p(c;W_{o:c})=\frac{e^{(W^T_{o:c} h^3)}}{e^{(\Sigma _1^c W^T_{o:c} h^3)}} \end{aligned}$$

where $C = 1,2$; denote the number of the class; $W_{o:c}$: is the class *c* weighting vector; $h^3$: are the output features from the last hidden layer, (third one), of the AE. In the last stage, the output probabilities of the shape, appearance and breath analysis networks, were combined together with the probabilities of the k-NN classier, and input into a softmax layer to estimate the fused classication decision. A leave-one-subject-out (LOSO) cross validation to classify the nodules for the 47 patients with both breath and CT data.

The larger patient cohort (467 patients) with 727 samples (413 benign and 314 malignant) was used to test methodology and classification accuracy of each of the imaging markers. Similarly, the breath analysis data from 504 patients were used to test the methodology and classification accuracy of using breath markers. Due to the large patient cohort, $75\%$ of these CT and breath data was used for training the AE network and $25\%$ data was used for validation. Classification accuracy using *C*4.5 algorithm, random forests, adaboost, SVM, and staked autoencoder with softmax were compared.

## Experimental results

The classification accuracy, sensitivity, and specificity for each of the different features combinations for the 47 patients is shown in Table 2. Nodule size had the least accuracy and sensitivity while shape and appearance features had the highest accuracy and sensitivity. For the patients for whom both breath and CT data were collected, the integration of all CT and breath markers using the CAD system resulted in accuracy, sensitivity, specificity, and AUC above $97\%$.

**Table 3:** Diagnosis accuracy in terms of accuracy, sensitivity, and specificity for various features groups (sample $\hbox {size} =47$ patients).

	Performance measures		
	Accuracy (%)	Sensitivity (%)	Specificity (%)
Size	61.19	29.73	100.00
Shape	89.55	89.19	90.00
Appearance	86.57	91.86	80.00
Breath analysis	75.99	71.43	80.56
Shape + size	91.04	89.19	93.33
Appearance + size	89.55	91.89	86.67
Shape + appearance	91.04	94.59	86.67
Shape + breath	89.55	89.19	90.00
Appearance + breath	88.06	91.89	83.33
Size + breath	79.10	72.97	86.67
Shape + size + breath	92.54	91.89	93.33
Shape + appearance + breath	92.65	94.74	90.00
Size + appearance + breath	92.54	94.59	90.00
Imaging features only	94.03	91.89	96.67
Combined features	97.87	97.30	100.00

In the larger patient cohort ($\hbox {breath} = 504$ patients, CT imaging = 467 patients), the accuracy, sensitivity, and specificity of size ($79.84\%$, $75.63\%$, and $83.59\%$), shape ($89.91\%$, $96.77\%$, and $84.80\%$), appearance ($89.91\%$, $93.55\%$, and $87.20\%$), and breath ($80.95\%$, $79.69\%$, and $82.26\%$) markers were similar to the values obtained with the CAD system for individual markers with the smaller patient cohort (Table 3). The stacked AE with softmax had the highest classification accuracy, sensitivity, and specificity amongst all tested classifiers. Table 4 shows our performance metrics for each part of the framework and the fused framework after the combination process using the LIDC dataset for validation. It also compares our framework performance with other frameworks^31–34^.

**Table 4:** Performance comparison between the different imaging modules and their combination using LIDC dataset and different systems used the same dataset.

	Performance measures		
	Accuracy (%)	Sensitivity (%)	Specificity (%)
Size	75.63	83.59	79.84
Shape	96.77	84.80	89.91
Appearance	93.55	87.20	89.91
Combined feature	93.55	91.20	92.20
Orozco et al.^31^	90.90	73.91	82.00
Wei et al.^32^	89.30	86.00	87.65
Costa et al.^33^	93.42	91.21	91.81
Xie et al.^34^	84.19	92.02	89.53

## Discussion

The results of this work demonstrates that combining both breath bio-marker and imaging data will significantly improve the accuracy, sensitivity, and specificity for clinical diagnosis of lung cancer. Currently, diagnosis of indeterminate pulmonary nodules requires documenting nodule growth for up to two years using multiple CT scans without percutaneous biopsy, which is cost prohibitive, increases patient radiation exposure, and delays final diagnosis. Definitive diagnosis of cancer is accomplished by invasive methods including needle biopsy or bronchoscopy. The primary innovation of the CAD system is that it integrates patient breath bio-marker data with image-based CT markers from a single CT scan and a single breath test to provide an accurate, robust, and more rapid diagnosis of small lung nodules. Additional innovations of the CAD system include identification and use of new image based markers (spherical harmonics and Gibbs energy). The shape and appearance analyses accounts for the prior growth rate of the nodule from a single CT scan and minimizes or obviates the need for serial CT scan to document growth or the use of invasive biopsies. In addition, the proposed approach will enable accurate tracking of nodule recession or progression, which may significantly shorten the two-year window to track the impact of therapeutic regimens on the growth of malignant nodules, and may also lead to a more definitive determination of the best cancer treatment for each patient. Current “gold-standard” methods cannot provide this quality of care in a cost-effective manner. Currently, patients are first assessed using X-rays, followed by CT scans due to insurance coverage limits. X-Rays cannot be used to diagnose early stage lung cancer. While CT scanning dramatically improved lung cancer detection and survival, it still has a $94\%$ false positive rate and requires repeat scans to document nodule growth in order to diagnose lung cancer. Currently, only  $35\%$ of lung cancer patients are detected at an early stage (Stage IA). In literature, the accuracy, sensitivity and specificity of diagnosis using various features from CT scans are  85–90%^12,14^, which is not adequate for diagnosis. Thus, the current clinical standard is to document growth rate of nodules by serial CT scans or use biopsy. Our proposed CAD system and framework significantly improves on the accuracy, sensitivity, and specificity ($>97\%$) by integrating both breath and CT markers, which will enhance the early detection by shortening the time for diagnosis, and consequently the survival rate. Importantly, our breath analysis technology is cost effective ($20\$$ per test) compared to X-Rays. Importantly, breath analysis alone offers  $80\%$ nodule classification accuracy. Moreover, among the other clinical bio-markers the breath test is chose to be integrated with the imaging markers as the organic compounds are volatile in nature, which make the concentration of these compounds higher in the breath compared to other markers (e.g., saliva, urine, and/or blood). In addition, the breath test gives an immediate result as the exhaled breath is collected directly to the bag where we used the mass spectrometry to analyze it. Most importantly, the breath analysis gives a local diagnosis for the lung compared to other bio-markers (e.g., the urine bio-markers will work better for detecting the tumors within the kidney). The CAD system framework is robust to loss of an individual marker, and is capable of integrating additional bio-marker data (eg. blood, saliva, urine etc.) to further improve accuracy. The CAD system currently considers 1098 features (1000 appearance features, 70 shape features, size, and 27 VOCs) for nodule classification. A three layered AE network was used to reduce it to 121 features (100 appearance features, 10 shape features, size, and 10 VOCs) that provided the highest discrimination to minimize computational cost and enable rapid classification. The sample size of patients with both breath and CT imaging data was limited ($n=47$) and thus a LOSO validation method was used. However, individual markers yielded a similar classification accuracy, sensitivity, and specificity with the larger retrospective patient cohort with $75\%$ training data set and $25\%$ test data set, validating the classification methodology and framework. The imaging markers of the CAD system were validated using data from 727 nodules from the LIDC database. While imaging data is available from other databases (eg. LUNA^35^, LUNGX^36^), the LIDC database was chosen specifically because it had a large patient cohort, and had a nodule malignancy score assigned by a team of radiologists to validate nodule classification. The LUNA database did not have a malignancy score and cannot be used for validating nodule diagnosis and the LUNGX database only consisted of 70 patients. Currently, there are no databases with both breath and imaging data other than our small patient cohort ($n=47$). The limitation of the CAD system is that although it can accurately classify nodules as malignant or benign, it could not differentiate between the different categories in each type (e.g. Lymphoma, Carcinoid, Sarcoma, Metastatic tumors, etc.). A larger clinical study will be needed to validate the CAD system for Food and Drug Administration approval. Despite these limitations, the CAD system and framework demonstrated the feasibility of a CAD system and framework for highly accurate lung cancer diagnosis using a single, inexpensive breath test and a single CT scan.

### Limitations

As with the majority of CAD systems, the design of the this system is subject to limitations that could be addressed in future research. First, the patients are enrolled to the study as they came to the clinic, which make the study had an inherent risk of selection bias, despite our inclusion criteria not having any demographic filters that might introduce bias. Second, the sample size of the patients that had both CT scans and breath test is small, which needs additional study that recruits more individuals to ensure that there are no significant covariables that may be influencing the data.

## Conclusion

This work presented a novel CAD system and framework for the diagnosis of pulmonary nodules by utilizing both imaging markers and breath bio-markers. The CAD system integrates patient breath bio-marker data with image-based CT markers obtained from a single CT scan and a single breath test to provide a highly accurate, rapid, cost-effective, and non-invasive diagnosis of small lung nodules.

## Data Availability

Materials, data, and associated protocols will be available to readers after the manuscript being accepted.
